# Inhibition of METTL14 Alleviates OGD/R‐Induced Neuronal Differentiation Injury in Neural Stem Cells Through Suppressing the Tp53inp2/Ptgs2 Axis: Potential Benefits for the Mouse MCAO Model

**DOI:** 10.1002/cns.71099

**Published:** 2026-09-01

**Authors:** Dan Hou, Yujie Hu, Tian Yun, Dan Yu, Guoshuai Yang

**Affiliations:** ^1^ Department of Neurology, Haikou Research Institute of Xiangya School of Medicine Central South University Haikou Hainan China

**Keywords:** cerebral ischemia‐reperfusion, m6A, METTL14, mitophagy, neural stem cell differentiation, Tp53inp2

## Abstract

**Background:**

Ischemic stroke (IS) is an acute cerebrovascular disease characterized by high morbidity and mortality, with limited current treatment options. Tumor protein p53‐inducible nuclear protein 2 (Tp53inp2) is known to be a positive regulator of autophagy under physiological conditions, but the mechanism of Tp53inp2 in IS remains unclear. In this study, we aimed to explore the mechanism of Tp53inp2 in IS.

**Methods:**

Primary neural stem cells (NSCs) were extracted and identified. An OGD/R cell model was constructed. Tp53inp2 was knocked down and rapamycin was added. A middle cerebral artery occlusion (MCAO) animal model was constructed, and then 5 μL of 5 × 10^5^ NSCs, either untreated or transfected with sh‐NC or sh‐Tp53inp2, were injected. Additionally, at the cellular level, Ptgs2 or Tp53inp2 was overexpressed, and METTL14 was knocked down.

**Results:**

Inhibition of Tp53inp2 mitigated OGD/R‐induced mitophagy and ROS levels in vitro. Moreover, inhibition of Tp53inp2 mediated neuronal differentiation of OGD/R treated NSCs by suppressing mitophagy. At the animal level, the transplantation of NSCs with a knockdown of Tp53inp2 increased neuronal differentiation, thereby alleviating the effects of MCAO and mitigating cognitive impairments in MCAO model mice. Ptgs2 was further screened and validated as a downstream target mediating the effect of Tp53inp2 on NSCs. At the cellular level, Tp53inp2 alleviated OGD/R‐induced mitophagy and ROS levels by regulating Ptgs2 expression. METTL14 could regulate Tp53inp2 expression by modulating the functional m6A modification sites on Tp53inp2 mRNA. Inhibition of METTL14 alleviated OGD/R‐induced mitophagy and the rise of ROS levels in NSCs by inhibiting the Tp53inp2/Ptgs2 axis.

**Conclusions:**

Inhibition of METTL14 alleviated OGD/R‐induced neuronal differentiation injury in NSCs by inhibiting the Tp53inp2/Ptgs2 axis. By elucidating the mechanisms involving Tp53inp2, Ptgs2, and METTL14, this research offers a foundation for developing new strategies to enhance neuronal differentiation and mitigate cognitive impairments in stroke patients.

## Graphical abstract

The graphical abstract highlights that ischemic stroke leads to increased METTL14 expression, which enhances Tp53inp2 mRNA stability through m^6^A modification. Elevated Tp53inp2 then promotes damaging mitophagy and ROS production by upregulating Ptgs2, ultimately impairing the neuronal differentiation of neural stem cells.

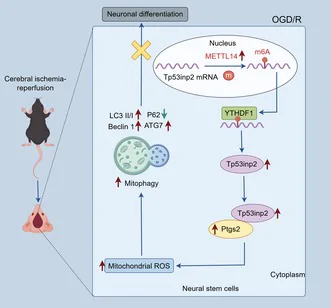

## 1 Introduction

Stroke is the second leading cause of death globally and a major contributor to long‐term disability [1]. Among the various types of stroke, ischemic stroke (IS), resulting from the blockage of blood vessels crucial for brain perfusion, is considered the most common, accounting for about 86% of all stroke cases [2]. Persistent ischemia in brain tissue can result in irreversible neuronal damage, leading to neurological dysfunction and significantly impacting the quality of life of patients [3]. At present, the lack of a complete understanding of the mechanisms behind neuronal damage in IS has led to a lack of US Food and Drug Administration‐approved therapies that can effectively enhance recovery from neurological damage caused by IS [4, 5]. Therefore, the treatment of IS remains a clinical challenge. Efforts to investigate the pathophysiological mechanisms and develop novel therapeutic strategies are critically needed.

Tumor protein p53‐inducible nuclear protein 2 (Tp53inp2) is an important positive regulator of autophagy under physiological conditions [6]. In mice, Tp53inp2 is encoded by Trp53inp2 [7]. Trp53inp2, which encodes the rate‐limiting factor in autophagy initiation, is the most highly induced autophagy‐related gene [8]. Recent studies have found that miR‐125b partially protects neurons from neuroinflammation and apoptosis induced by abnormal activation of p53 networks during ischemia–reperfusion injury by downregulating Tp53inp1 [9]. Tp53inp2 enhances osteogenic differentiation of adipose‐derived stem/stromal cells through activation of Wnt/β‐catenin signaling [10]. In addition, IS can alter the brain N6‐methyladenosine (m6A) epigenetic transcriptome, which may have functional significance in the pathophysiology of IS [11]. It has been reported that m6A modification mediated by the demethylase FTO regulates the cytoplasmic expression of Tp53inp2 and promotes autophagy of leukemia cells [12]. The role and mechanism of Tp53inp2 in IS have not been previously documented, and it is uncertain whether Tp53inp2 undergoes m6A methylation in the context of IS.

During IS, autophagy is triggered in different types of brain cells, including neurons, glial cells, and microvascular cells [13]. Mitochondria are the primary targets of hypoxic/ischemic injury [14]. Mitophagy is a form of selective autophagy that involves the removal of damaged or dysfunctional mitochondria through specific autophagic mechanisms to prevent the excessive production of reactive oxygen species (ROS) and consequent cell death [15]. Given the polarized morphology of neurons, the regulation of mitophagy varies across different neuronal compartments. Following ischemia‐reperfusion injury, mitochondria in the axons are degraded by autophagy in the cell bodies [16]. In addition, reperfusion induces oxidative damage and cell death through the production of mitochondrial ROS [17]. KLF2 has been reported to regulate the differentiation of dental pulp‐derived stem cells by inducing mitophagy and altering mitochondrial metabolism [18]. However, whether Tp53inp2 is modified by m6A methylation in IS and affects mitophagy and the metabolism of neural stem cells (NSCs), thereby affecting NSC differentiation, remains unclear.

Building on the aforementioned background information, this study aims to explore the role of Tp53inp2 in IS through a combination of in vivo and in vitro experiments. We hypothesized that m6A‐modified Tp53inp2 may affect cognitive impairment in mice with cerebral ischemia‐reperfusion by regulating mitophagy and mediating stem cell differentiation. Our study may offer novel ideas and insights into the treatment of IS.

## 2 Materials and Methods

### 2.1 Study Design

This study first isolated primary NSCs from the brains of neonatal mice and identified them. Subsequently, an oxygen‐glucose deprivation/reperfusion (OGD/R) cell model was constructed to simulate ischemic conditions, and Tp53inp2 was knocked down using shRNA technology. Rapamycin, an autophagy activator, was also added to investigate its effects on NSC differentiation. Additionally, a middle cerebral artery occlusion (MCAO) model was established in mice, and NSCs transfected with sh‐NC or sh‐Tp53inp2 (5 × 10^5^ cells/5 μL) were transplanted into the brains of MCAO mice to evaluate their effects on ischemic injury and cognitive impairments. At the cellular level, the effects of overexpressing Ptgs2 or Tp53inp2 and knocking down METTL14 on OGD/R‐induced mitophagy and ROS levels were examined. The regulatory role of METTL14 in Tp53inp2 m6A modification was verified through m6A‐RNA immunoprecipitation (RIP) and RIP experiments, and the interaction between Tp53inp2 and Ptgs2 was confirmed using co‐immunoprecipitation (Co‐IP) assays. Ultimately, the study revealed the molecular mechanism by which METTL14 inhibition alleviates OGD/R‐induced NSC differentiation injury through the Tp53inp2/Ptgs2 axis. Table S1 shows the primers used in this study. Further details about the methods are available in the Data S1.

### 2.2 Statistical Analysis

Statistical analysis was carried out using GraphPad Prism 8.0 software. Measurement data were presented as the mean ± standard deviation. Student's *t*‐test was applied for comparisons between two groups, while one‐way or two‐way analysis of variance (ANOVA) was utilized for comparisons among multiple groups, followed by Tukey's post hoc test. *p* < 0.05 was considered statistically significant.

## 3 Results

### 3.1 Inhibition of Tp53inp2 Alleviated OGD/R‐Induced Mitophagy and ROS Levels, Mediating Neuronal Differentiation of OGD/R‐Treated NSCs Through Repression of Mitophagy

**FIGURE 1:**
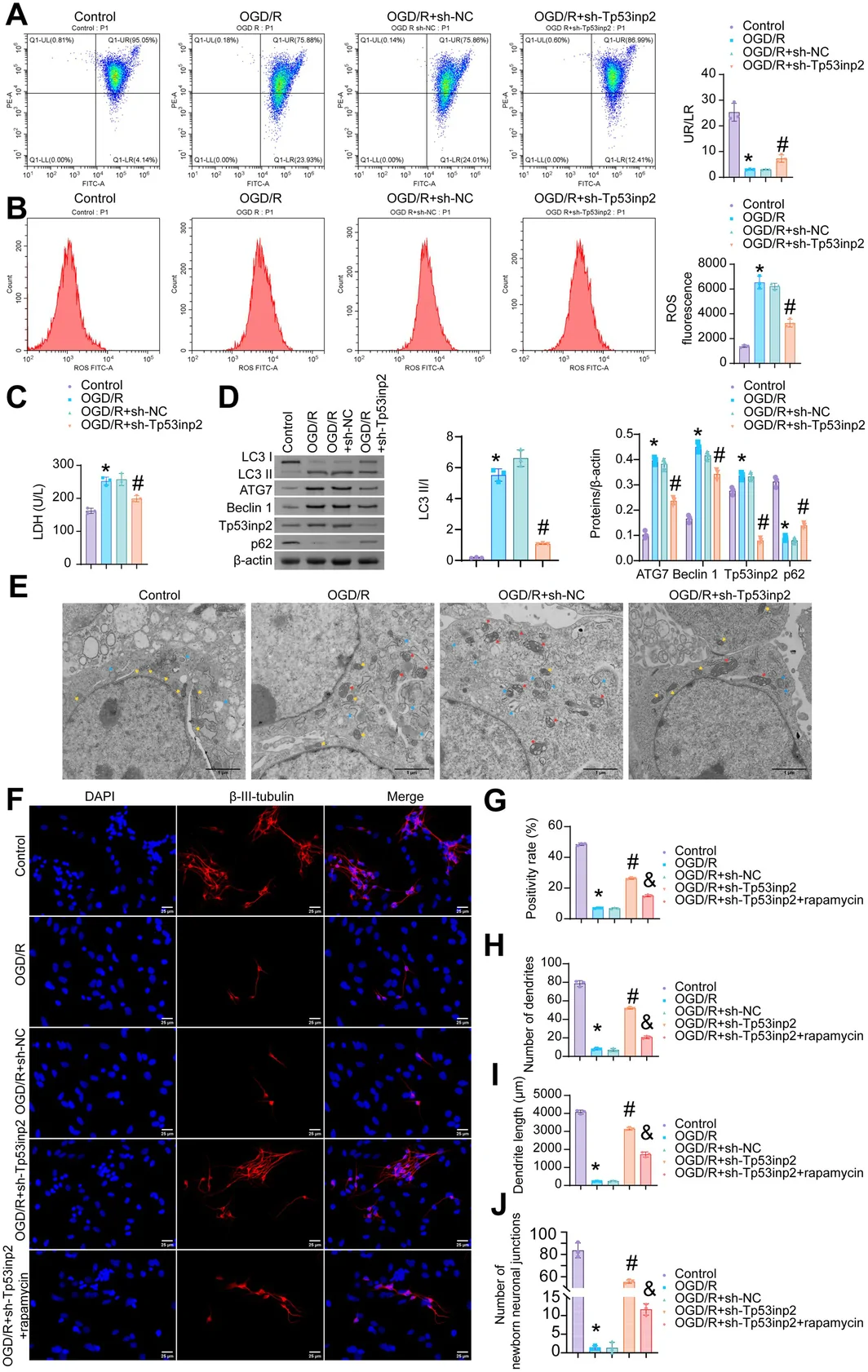
Inhibition of Tp53inp2 alleviated OGD/R‐induced mitophagy and ROS levels, mediating neuronal differentiation of OGD/R‐treated NSCs through repression of mitophagy. (A) LDH level. (B) Flow cytometry analysis of mitochondrial membrane potential. (C) Mitochondrial ROS levels were monitored by flow cytometry. (D) Western blot analysis of Tp53inp2 and autophagy‐related proteins LC3 II/I, ATG7, Beclin 1, and p62. (E) Changes in mitochondria and autophagosomes were observed using TEM. Normal mitochondria are indicated by yellow arrows, damaged mitochondria are indicated by red arrows, and autophagosomes are indicated by blue arrows. Scale bar = 1 μm. (F) IF of β‐III‐tubulin expression. Scale bar = 25 μm (400 ×). (G) Statistics on the positive rate of β‐III‐tubulin. (H) Number of dendrites statistics. (I) Total dendrite length statistics. (J) Statistics on the number of junctions of newborn neurons. *p < 0.05 versus Control, #*p* < 0.05 versus OGD/*R* + sh‐NC, & *p* < 0.05 versus OGD/*R* + sh‐Tp53inp2. For the cell experiment, *n* = 3.

Studies have shown that Tp53inp2 could induce autophagy in bovine adipocytes [19], and OGD/R could induce autophagy in NSCs [20]. However, whether Tp53inp2 contributes to mitophagy in OGD/R‐induced NSCs has not been investigated. First, we isolated and identified primary NSCs. Figure S1A showed an optical image of the primary NSCs. IF identification showed that Nestin was positively expressed (Figure S1B), indicating that we successfully extracted primary NSCs. Next, we interfered with Tp53inp2. In the sh‐Tp53inp2 group, Tp53inp2 expression was markedly decreased compared with the sh‐NC group (Figure S1C), confirming successful knockdown of Tp53inp2. Next, we constructed an OGD/R cell model. Compared with the control group, LDH level, ROS level, and Tp53inp2 level were elevated in the OGD/R group, while mitochondrial membrane potential was decreased. After interference with Tp53inp2, LDH level, ROS level, and Tp53inp2 expression were decreased, but mitochondrial membrane potential was increased (Figure 1A–1D). In addition, in the OGD/R group, the levels of autophagy‐related proteins LC3 II/I, ATG7, and Beclin 1 levels were elevated, while the level of p62 was reduced compared with the control group. After interference with Tp53inp2, the levels of LC3 II/I, ATG7, and Beclin 1 decreased, and the levels of p62 elevated (Figure 1D). TEM showed that the mitochondrial matrix in the control group was dense, the cristae were neatly arranged, and no signs of autophagy were detected. In the OGD/R group, mitochondria displayed swelling, with partially broken or disorganized cristae. Some double‐membrane‐bound compartments containing cytoplasmic substances (autophagosomes) could be seen. Interference with Tp53inp2 reversed OGD/R‐induced changes (Figure 1E). These results suggested that inhibition of Tp53inp2 alleviated OGD/R‐induced mitophagy and ROS levels. It has been documented that Tp53inp2 can induce autophagy during the initial phase of bovine adipocyte differentiation and promote adipocyte differentiation through the modulation of autophagy [19]. However, whether Tp53inp2 mediates neuronal differentiation of NSCs through mitophagy and changes in ROS levels has not been reported. Next, we added the autophagy activator rapamycin to treat the primary NSCs. In the OGD/R group, β‐III‐tubulin expression was decreased compared with the control group. Following interference with Tp53inp2, β‐III‐tubulin level elevated. However, with the addition of rapamycin, β‐III‐tubulin expression decreased (Figure 1F,G). In addition, compared with the control group, the number of dendrites, total dendrite length, and the number of new neuronal junctions were all suppressed in the OGD/R group. After interference with Tp53inp2, the number of dendrites, the total dendrite length, and the number of new neuronal junctions all increased. After the addition of rapamycin, the number of dendrites, the total dendrite length, and the number of neuronal junctions were reduced (Figure 1H–1J). These results suggested that knockdown of Tp53inp2 mediated the neuronal differentiation of OGD/R treated NSCs by repressing mitophagy.

### 3.2 The Transplantation of NSCs With Knockdown of Tp53inp2 Increased Neuronal Differentiation to Alleviate MCAO In Vivo

**FIGURE 2:**
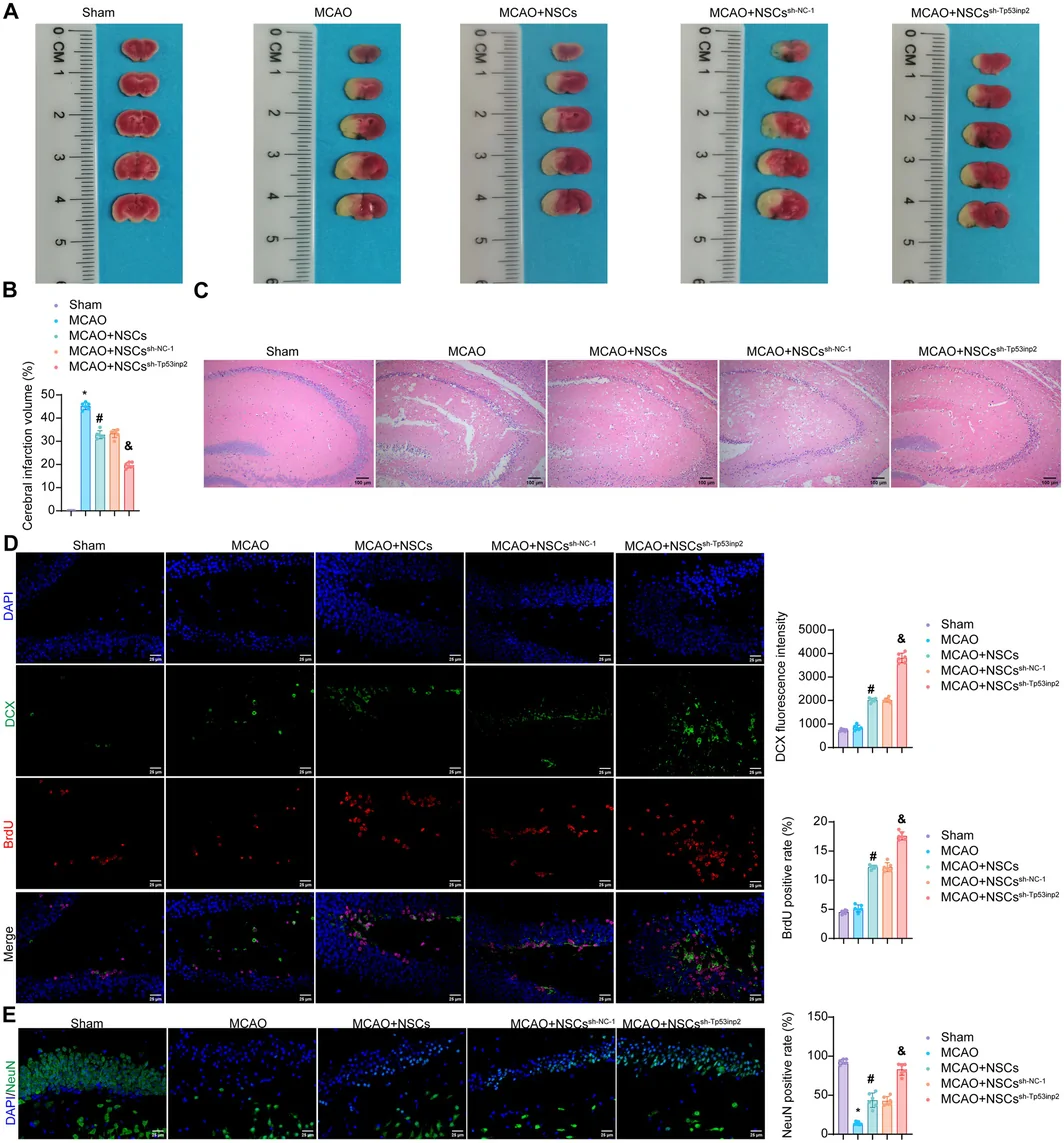
The transplantation of NSCs with knockdown of Tp53inp2 increased neuronal differentiation to alleviate MCAO in vivo. (A) Representative TTC stained brain sections. (B) TTC staining of the volume of cerebral infarction. (C) HE staining was performed to detect brain injury. Scale bar = 100 μm (100 ×). (D) IF staining of DCX and BrdU in the hippocampus. (E) IF staining of NeuN in the hippocampus. Scale bar = 25 μm (400×). *p < 0.05 versus Sham, #*p* < 0.05 versus MCAO, & *p* < 0.05 versus MCAO + NSCs^sh‐NC‐1^. For animal experiments, *n* = 6.

Furthermore, we constructed the MCAO model and knocked down Tp53inp2. Figure 2A showed representative TTC‐stained brain sections. Compared with the sham group, the MCAO group exhibited an increased cerebral infarct volume. After the NSC transplantation, the cerebral infarct volume decreased. Further knockdown of Tp53inp2 led to a further reduction in the cerebral infarct volume (Figure 2B). HE staining revealed that transient cerebral ischemia followed by referfusion in mice led to increased neuronal damage in the hippocampal region. After NSC transplantation, neuronal damage was reduced. Furthermore, transplantation of NSCs transfected with sh‐Tp53inp2 transfection resulted in a further reduction in neuronal damage (Figure 2C). Furthermore, compared with the sham group, BrdU and DCX levels in the MCAO group showed an upward trend, but there was no significant change. After NSC transplantation, the levels of BrdU and DCX increased. Furthermore, transplantation of NSCs transfected with sh‐Tp53inp2 led to a further increase in BrdU and DCX levels (Figure 2D). Additionally, compared with the sham group, the NeuN‐positive rate was significantly reduced in the MCAO group. After NSC transplantation, the NeuN‐positive rate increased. Furthermore, transplantation of NSCs transfected with sh‐Tp53inp2 transfection led to a further increase in the NeuN positive rate (Figure 2E). These results indicated that the transplantation of NSCs with knockdown of Tp53inp2 increased neuronal differentiation to alleviate MCAO in vivo.

To verify the overexpression efficiency of Tp53inp2, Western blotting was utilized to detect Tp53inp2 protein levels. The results showed that compared with the oe‐NC group, Tp53inp2 protein expression was significantly upregulated in the oe‐Tp53inp2 group (Figure S2A), confirming the successful construction of the overexpression vector and its transfection efficiency. Flow cytometry was employed to detect mitochondrial ROS levels in each group. The results showed that compared with the oe‐NC group, the oe‐Tp53inp2 group exhibited no significant change in mitochondrial ROS levels; the sh‐Tp53inp2 group showed a slight increase in mitochondrial ROS levels compared with the sh‐NC group, but the difference was not statistically significant (Figure S2B). These results indicated that altering Tp53inp2 expression has no significant effect on mitochondrial ROS levels. Western blot analysis of autophagy marker protein expression showed that compared with the oe‐NC group, the oe‐Tp53inp2 group exhibited a slight increase in the LC3 II/I ratio, ATG7 and Beclin 1 expression, and a slight decrease in p62 expression. Compared with the sh‐NC group, the sh‐Tp53inp2 group showed a slight decrease in the LC3 II/I ratio, ATG7 and Beclin 1 expression, and a slight increasing trend in p62 expression. However, none of these changes reached statistical significance (Figure S2C). These findings suggested that altering Tp53inp2 expression has a limited effect on cellular autophagy levels. Analysis of β‐III‐tubulin expression showed that compared with the oe‐NC group, the oe‐Tp53inp2 group exhibited a slight increase in the percentage of β‐III‐tubulin‐positive cells; compared with the sh‐NC group, the sh‐Tp53inp2 group showed a slight decrease in the percentage of β‐III‐tubulin‐positive cells; however, the differences were not statistically significant (Figure S2D,E). Further quantitative analysis of neuronal differentiation‐related indicators showed that compared with the oe‐NC group, the oe‐Tp53inp2 group exhibited slight increases in the number of dendrites, total dendrite length, and the number of new neuronal junctions; compared with the sh‐NC group, the sh‐Tp53inp2 group showed slight decreases in the total dendrite length and the number of new neuronal junctions, but none of these differences reached statistical significance (Figure S2F–H). In summary, neither overexpression nor knockdown of Tp53inp2 had significant effects on mitochondrial ROS, autophagy, or neuronal differentiation.

### 3.3 The Transplantation of NSCs With Knockdown of Tp53inp2 Mitigated Cognitive Impairments in MCAO Model Mice

**FIGURE 3:**
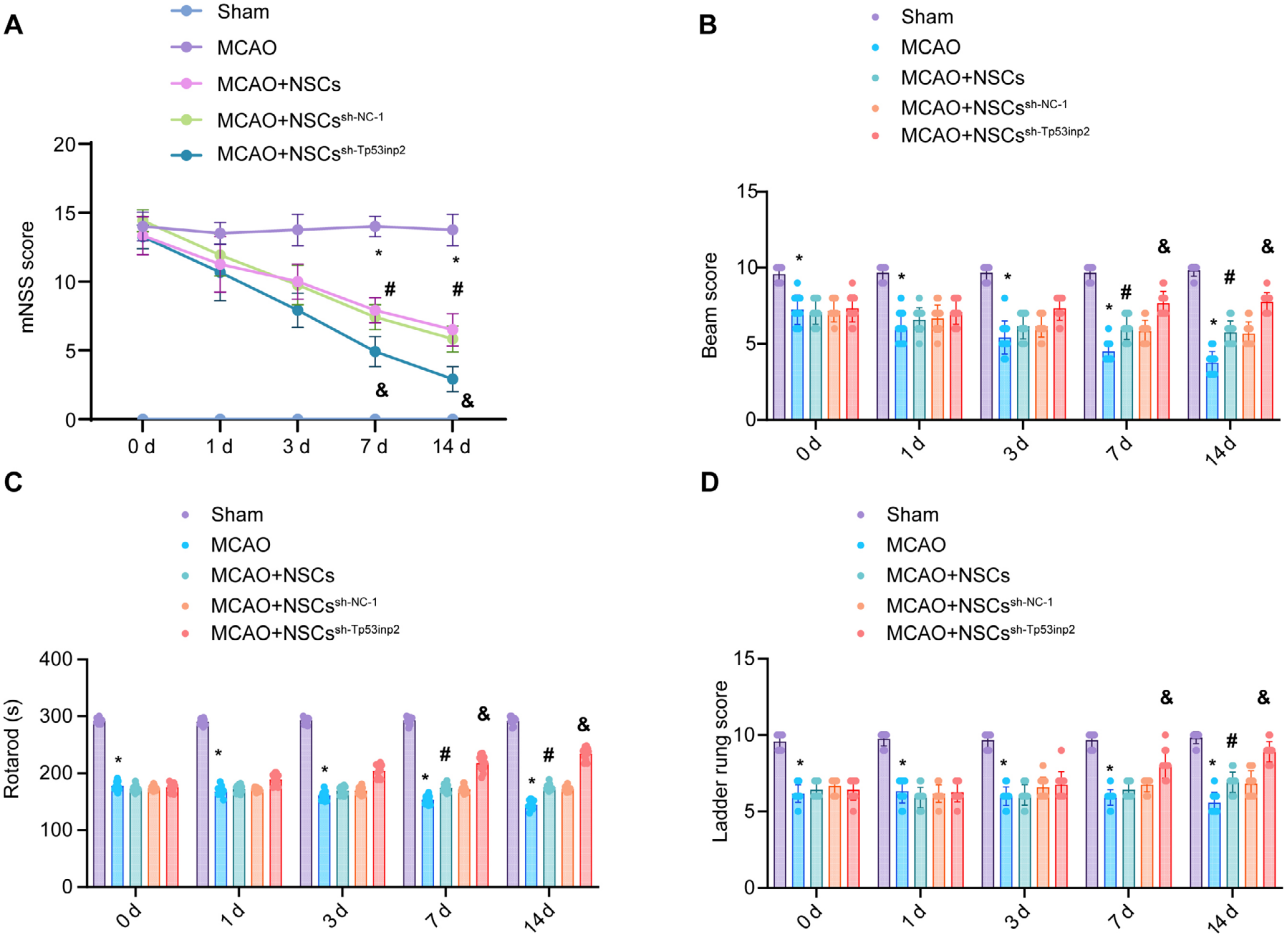
The transplantation of NSCs with knockdown of Tp53inp2 mitigated cognitive impairments in MCAO model mice. (A–D) mNSS and behavioral test results (the balance beam, rotarod tests, and ladder lung) at 0, 1, 3, 7, and 14 days after treatment. *p < 0.05 versus Sham, #*p* < 0.05 versus MCAO, & *p* < 0.05 versus MCAO + NSCs^sh‐NC‐1^. For animal experiments, *n* = 6.

After 14 days of treatment, neurological function was assessed through the mNSS and behavioral tests, including the balance beam, rotarod, and ladder‐walk tests. The results indicated that NSCs began to exert their effects 7 days after the MCAO model, as evidenced by a decrease in the mNSS score and increases in the Beam score, rotarod duration, and ladder rung score. By 14 days post‐treatment, transplantation of NSCs transfected with sh‐Tp53inp2 transfection showed significantly better outcomes than transplantation of NSCs alone (Figure 3A–D). These results suggested that the transplantation of NSCs with knockdown of Tp53inp2 mitigated cognitive impairments in MCAO model mice.

### 3.4 Downstream Target Screening for the Effect of Tp53inp2 on NSCs

**FIGURE 4:**
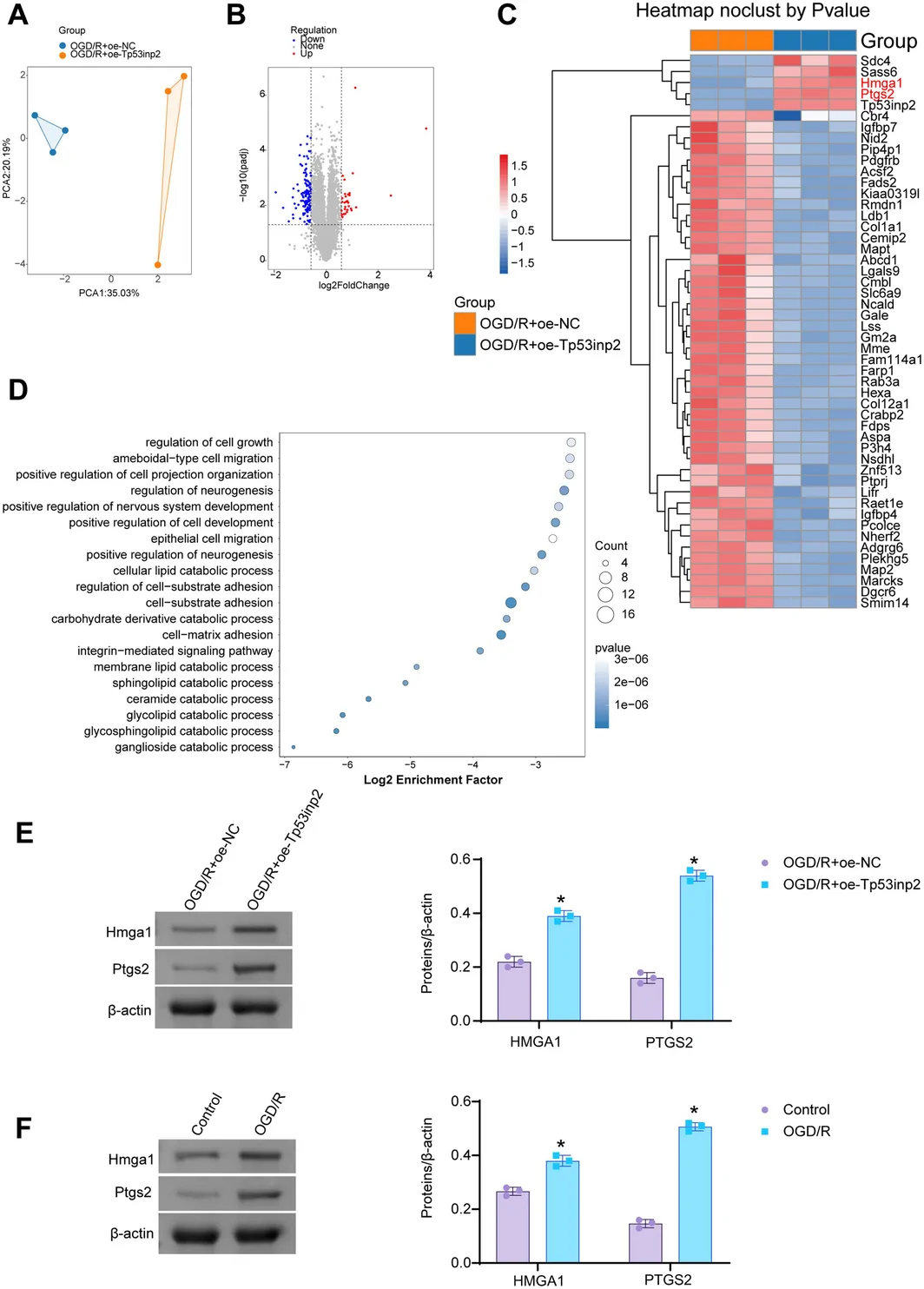
Downstream target screening for the effect of Tp53inp2 on NSCs. (A) PCA diagram. (B and C) Volcano and Heat maps of differentially expressed proteins. (D) KEGG enrichment bubble map. (E) Western blot analysis of up‐regulated proteins Ptgs2 and Hmga1. *p < 0.05 versus oe‐NC. (F) Western blot analysis of up‐regulated proteins Ptgs2 and Hmga1. *p < 0.05 versus Control. For the cell experiment, *n* = 3.

Next, we screened downstream targets for the effects of Tp53inp2 on NSCs. Figure 4A showed the PCA diagram. The sample composition of the two groups was significantly different. Volcano plots and heat maps showed upregulated and downregulated proteins (Figure 4B,C). KEGG analysis revealed that the differentially expressed proteins were mainly enriched in the regulation of cell growth, ameboidal‐type cell migration, positive regulation of cell projection organization, cell‐substrate adhesion, and regulation of neurogenesis (Figure 4D). Previous research has shown that overexpression of Hmga1 can reduce mitochondrial membrane potential, leading to mitochondrial dysfunction, and increase mitochondrial ROS levels, further inducing cell apoptosis [21]. In one study, the mitochondrial membrane potential of cells was detected using the JC‐1 fluorescent probe, and upregulation of Ptgs2 was found to be associated with a decrease in mitochondrial membrane potential, suggesting that Ptgs2 activity may lead to mitochondrial dysfunction by increasing oxidative stress levels within the cell [22]. Compared with the OGD/*R* + oe‐NC group, Ptgs2 and Hmga1 expression levels in the OGD/R + oe‐Tp53inp2 group were increased, among which Ptgs2 expression showed the greatest increase (Figure 4E). These findings suggested that Tp53inp2 could enhance the expression of Ptgs2 and Hmga1. Subsequent experimental validation revealed that, compared with the control group, Ptgs2 and Hmga1 expression levels were elevated in the OGD/R group. Notably, Ptgs2 expression exhibited the greatest increase (Figure 4F). Therefore, we chose Ptgs2 for further study.

### 3.5 Knockdown of Tp53inp2 Alleviated OGD/R‐Induced Mitophagy and ROS Levels by Regulating Ptgs2 Expression in NSCs

**FIGURE 5:**
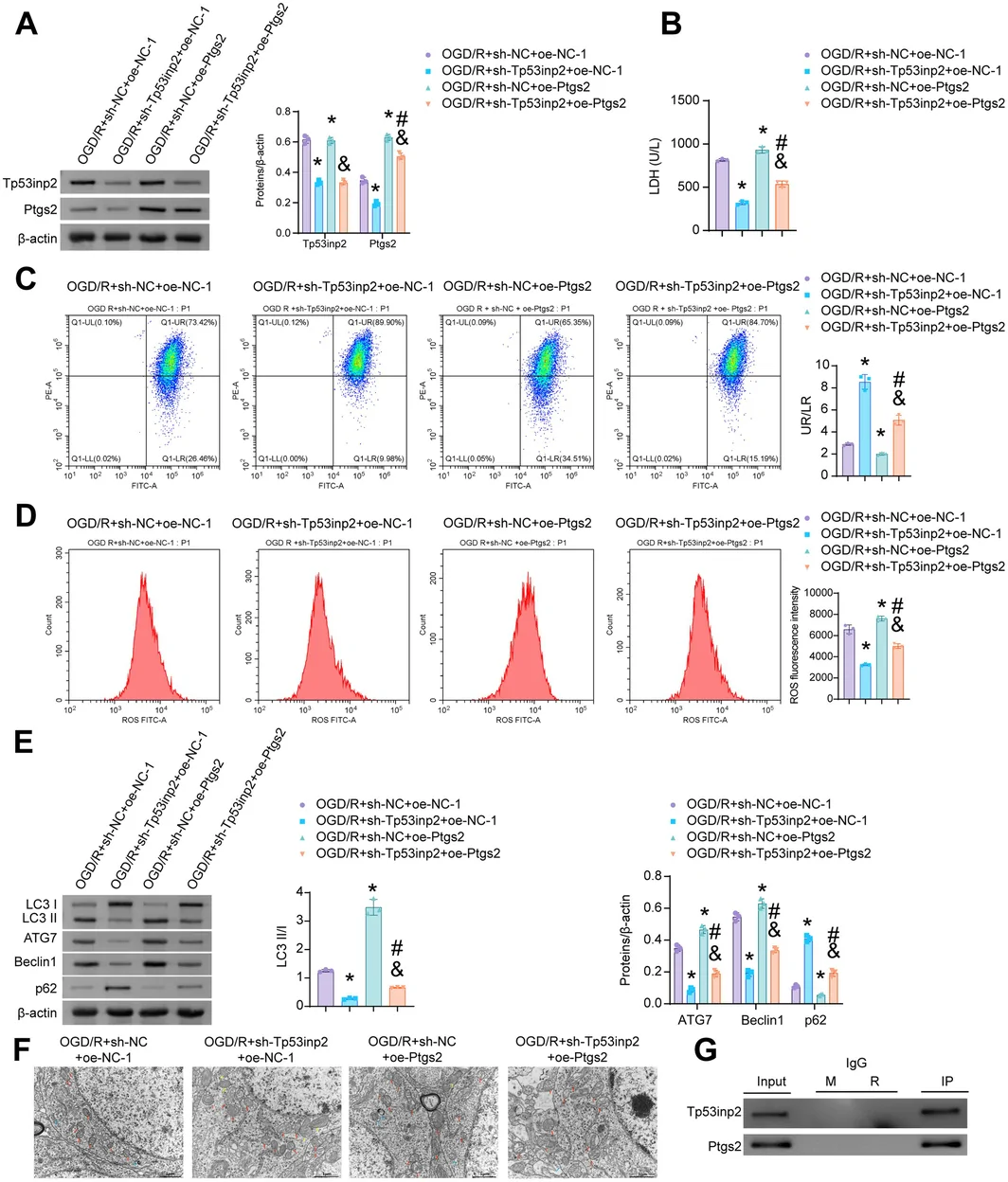
Knockdown of Tp53inp2 alleviated OGD/R‐induced mitophagy and ROS levels by regulating Ptgs2 expression in NSCs. (A) Western blot analysis of Tp53inp2 and Ptgs2 levels. (B) LDH level. (C) Flow cytometry analysis of mitochondrial membrane potential. (D) Mitochondrial ROS levels were determined through flow cytometry. (E) Western blot analysis of Tp53inp2 and autophagy‐related proteins LC3 II/I, ATG7, Beclin 1, and p62. (F) Changes in mitochondria and autophagosomes were assessed by TEM. Normal mitochondria are indicated by yellow arrows, damaged mitochondria are indicated by red arrows, and autophagosomes are indicated by blue arrows. Scale bar = 1 μm. (G) Co‐IP detection of whether Tp53inp2 binds to Ptgs2 protein. *p < 0.05 versus OGD/*R* + sh‐NC + oe‐NC‐1, #*p* < 0.05 versus OGD/*R* + sh‐Tp53inp2 + oe‐NC‐1, & *p* < 0.05 versus OGD/*R* + sh‐NC + oe‐Ptgs2. For the cell experiment, *n* = 3.

Next, we further explored the mechanism of Tp53inp2 and Ptgs2 in NSCs. Compared with the OGD/*R* + sh‐NC + oe‐NC‐1 group, Tp53inp2 and Ptgs2 expression levels were reduced in the OGD/*R* + sh‐Tp53inp2 + oe‐NC‐1 group, and Ptgs2 expressions were increased in the OGD/*R* + sh‐NC + oe‐Ptgs2 group. Additionally, compared with the OGD/*R* + sh‐Tp53inp2 + oe‐NC‐1 group, Ptgs2 expression was elevated in the OGD/*R* + sh‐Tp53inp2 + oe‐Ptgs2 group (Figure 5A). Moreover, in the OGD/R model, sh‐Tp53inp2 reduced LDH and ROS levels and increased mitochondrial membrane potential, indicating a protective effect against OGD/R‐induced damage. However, oe‐Ptgs2 alone increased LDH and ROS levels and decreased mitochondrial membrane potential, worsening OGD/R effects. Notably, when sh‐Tp53inp2 was combined with oe‐Ptgs2, it reversed the detrimental effects of oe‐Ptgs2, reducing LDH and ROS levels and increasing mitochondrial membrane potential (Figure 5B–D). Furthermore, sh‐Tp53inp2 decreased LC3 II/I, ATG7, and Beclin 1 levels and increased p62 levels, suggesting reduced autophagy. In contrast, oe‐Ptgs2 increased LC3 II/I, ATG7, and Beclin 1 levels and decreased p62 levels, indicating enhanced autophagy. The combination of sh‐Tp53inp2 and oe‐Ptgs2 led to an increase in LC3 II/I, ATG7, and Beclin 1 levels and a decrease in p62 levels, further supporting the role of Tp53inp2 in regulating autophagy through Ptgs2 (Figure 5E). TEM showed that sh‐Tp53inp2 mitigated OGD/R‐induced mitochondrial swelling and disorganization, while oe‐Ptgs2 exacerbated these effects (Figure 5F). Finally co‐IP confirmed that Tp53inp2 bound to the Ptgs2 protein (Figure 5G). Taken together, knockdown of Tp53inp2 alleviated OGD/R‐induced mitophagy and ROS levels by regulating Ptgs2 expression in NSCs.

### 3.6 METTL14 Promoted Tp53inp2 m6A Levels in NSCs and Inhibition of METTL14 Alleviated OGD/R‐Induced Mitophagy and the Rise of ROS Levels in NSCs by Inhibiting the Tp53inp2/Ptgs2 Axis

**FIGURE 6:**
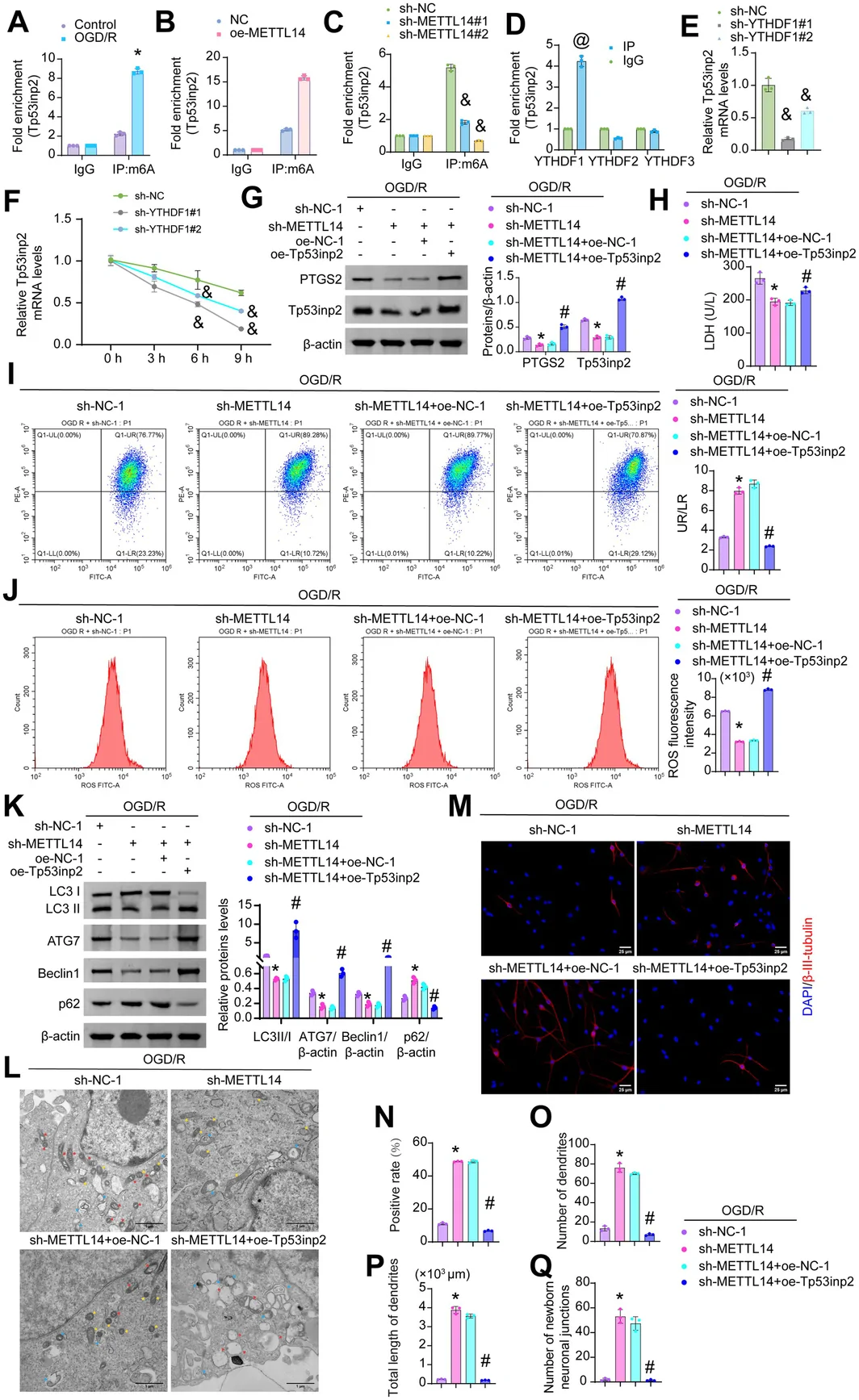
METTL14 promoted Tp53inp2 m6A levels in NSCs and inhibition of METTL14 alleviated OGD/R‐induced mitophagy and the rise of ROS levels in NSCs by inhibiting the Tp53inp2/Ptgs2 axis. (A) m6A‐RIP detection of the Tp53inp2 m6A level of NSCs. (B) m6A‐RIP detection of regulatory relationship between Tp53inp2 m6A level and METTL14. #*p* < 0.05 versus NC. (C) m6A‐RIP detection of Tp53inp2 m6A level of NSCs. & *p* < 0.05 versus sh‐NC. (D) RIP detection of whether YTHDF1, 2, 3 binds to Tp53inp2. (E) Tp53inp2 expression was assessed by qRT‐PCR. (F) Tp53inp2 mRNA stability was detected after actinomycin D treatment. & *p* < 0.05 versus sh‐NC. (G) Western blot analysis of Tp53inp2 and Ptgs2 levels. (H) LDH level. (I) Flow cytometry analysis of mitochondrial membrane potential. (J) Mitochondrial ROS levels were monitored by flow cytometry. (K) Western blot analysis of autophagy‐related proteins LC3 II/I, ATG7, Beclin 1, and p62. (L) Changes of mitochondria and autophagosomes determined by TEM. Normal mitochondria are indicated by yellow arrows, damaged mitochondria by red arrows, and autophagosomes by blue arrows. Scale bar = 1 μm. (M) β‐III‐tubulin expression was measured by IF. Scale bar = 25 μm (400×). (N) Statistics on the positive rate of β‐III‐tubulin. (O) Number of dendrites statistics. (P) Total dendrite length statistics. Q. Statistics on the number of junctions of newborn neurons. *p < 0.05 versus OGD/*R* + sh‐NC‐1, #*p* < 0.05 versus OGD/*R* + sh‐METTL14 + oe‐NC‐1. For the cell experiment, *n* = 3.

It has been reported that m6A modification mediated by demethylase FTO regulates cytoplasmic expression of Tp53inp2 and promotes autophagy of leukemia cells [12]. In addition, KLF2 regulates the differentiation of pulp‐derived stem cells by inducing mitophagy and altering mitochondrial function [18]. However, whether Tp53inp2 is methylated by m6A in MCAO and affects mitophagy and the function of NSCs and thus NSC differentiation remains unclear. To identify potential m6A modification sites on Tp53inp2 mRNA, the SRAMP online tool was used to analyze the Tp53inp2 sequence. SRAMP employs a random forest machine learning framework, integrating position‐specific nucleotide sequence patterns, k‐nearest neighbor information, and position‐independent dinucleotide pair spectrum features for prediction [23]. The prediction results revealed four high‐confidence m6A modification sites on Tp53inp2 mRNA, located at adenine (A) residues at positions 57, 63, 3755, and 4194, respectively (Figure S3A). The sites at positions 57 and 63 are spatially close and lie within the same predicted region. All these sites conform to the canonical DRACH (D = A/G/U, *R* = A/G, H = A/C/U) m6A consensus motif. Secondary structure analysis indicated that these m6A sites are all located in looped, unpaired regions of the RNA molecule, a structural feature that facilitates recognition and modification by the m6A methyltransferase complex (e.g., METTL3/METTL14) (Figure S3B). Then, we detected the Tp53inp2 m6A level of the NSCs using m6A‐RIP. Compared with the control group, the Tp53inp2 m6A level in OGD/R group was facilitated (Figure 6A). Compared with the control group, METTL14 levels were increased in OGD/R group, whereas there was no significant change in METTL16 levels (Figure S3C). Since the change in METTL14 expression was the most significant, we chose METTL14 for the follow‐up study. m6A‐RIP further verified the regulatory relationship between the Tp53inp2 m6A level and METTL14. Compared with the NC group, the Tp53inp2 m6A level was increased in oe‐METTL14 group (Figure 6B). Additionally, compared with the sh‐NC group, METTL14 expression decreased in both the sh‐METTL14#1 and sh‐METTL14#2 groups. The knockdown effect was more pronounced in the sh‐METTL14#2 group, as shown in Figure S3D,E, confirming successful knockdown of METTL14. Tp53inp2 expression was suppressed in sh‐METTL14#1 and sh‐METTL14#2 groups compared with the sh‐NC group (Figure S3F,G).

To experimentally validate the m6A modification sites predicted by bioinformatics, MeRIP‐qPCR was performed to detect the m6A modification level of Tp53inp2 mRNA. Specific primers were designed based on the four high‐confidence sites predicted by SRAMP, covering the RNA regions containing these sites (because positions 57 and 63 are close to each other, one primer pair was designed for that region; one primer pair was designed for the remaining two sites). The experimental groups were as follows: m6A‐1 group (covering the region of positions 57/63); m6A‐2 group (covering the region of position 3755); and m6A‐3 group (covering the region of position 4194). MeRIP‐qPCR results showed that, compared with the IgG control group, the m6A antibody exhibited significant enrichment in all predicted RNA regions (Figure S3H). Specifically, the enrichment folds of the m6A‐1, m6A‐2, and m6A‐3 groups were significantly different from those of the IgG group, confirming that m6A modifications indeed exist on Tp53inp2 mRNA in these predicted regions. Among these groups, the m6A‐3 group displayed the most significant difference. The enrichment fold of each region was consistent with the distribution of high‐confidence sites predicted by SRAMP, thereby validating the accuracy of the bioinformatics predictions. To further verify the contribution of specific m6A sites to the methylation of Tp53inp2 mRNA, site‐directed mutagenesis was performed on key m6A modification sites. Following established methods, the adenine (A) at the predicted sites was mutated to other nucleotides to disrupt the DRACH motif [24]. The mutagenesis strategy was as follows: Mut1 and Mut2 contained an A‐to‐G and an A‐to‐T mutation at position 4194, respectively (Figure S3I). The mutation schematic diagram shows that single‐base substitution effectively eliminates the modification potential of the target m6A sites. MeRIP‐qPCR was used to validate the mutation effects, with the following experimental groups: WT group (wild‐type, IgG control), MUT‐1 group (Mut1 mutant, m6A antibody IP), and MUT‐2 group (Mut2 mutant, m6A antibody IP). The results showed that, the m6A antibody significantly enriched Tp53inp2 mRNA in the WT group, while the enrichment levels in both MUT‐1 and MUT‐2 mutant groups were substantially reduced, approaching the IgG background level (Figure S3J). These findings indicated that site‐directed mutations successfully disrupted the corresponding DRACH motifs, resulting in an almost complete abolition of m6A modification at these sites, and confirmed that the 4194 site serves as a critical functional site for m6A modification of Tp53inp2 mRNA. m6A‐RIP showed reduced Tp53inp2 m6A levels in the sh‐METTL14#1 and sh‐METTL14#2 groups compared with the sh‐NC (Figure 6C). RIP further detected whether YTHDF1, YTHDF2, and YTHDF3 were combined with Tp53inp2. The results showed that only YTHDF1 combined with Tp53inp2 (Figure 6D). In addition, we have interfered with YTHDF1. Compared with the sh‐NC group, the expression of YTHDF1 in the sh‐YTHF1#1 group and sh‐YTHDF1#2 group decreased, and the effect of sh‐YTHDF1#1 was more obvious (Figure S3K,L), indicating that we successfully knocked down YTHDF1. Compared with the sh‐NC group, Tp53inp2 expression in sh‐YTHDF1#1 and sh‐YTHDF1#2 groups decreased (Figure 6E). Finally, Tp53inp2 mRNA stability was detected after actinomycin D treatment. Tp53inp2 mRNA levels in the sh‐YTHDF1#1 and sh‐YTHDF1#2 groups were decreased than those in the sh‐NC group (Figure 6F). Our data support the role of YTHDF1 in the m6A‐dependent regulation of Tp53inp2. These results revealed that METTL14 could regulate Tp53inp2 expression by modulating the functional m6A modification sites on Tp53inp2 mRNA. Furthermore, we interfered with METTL14 and overexpressed Tp53inp2 to explore the mechanism between METTL14 and Tp53inp2. Compared with the OGD/R + sh‐NC‐1 group, Tp53inp2 and Ptgs2 levels were reduced in OGD/*R* + sh‐METTL14 group. Following overexpression of Tp53inp2, both Tp53inp2 and Ptgs2 expressions promoted, as illustrated in Figure 6G. Compared with the OGD/R + sh‐NC‐1 group, LDH and ROS levels were suppressed and mitochondrial membrane potential was increased in the OGD/R + sh‐METTL14 group. Following overexpression of Tp53inp2, LDH and ROS levels were increased and mitochondrial membrane potential was decreased as shown in Figure 6H–J. As shown in Figure 6K, compared with the OGD/R + sh‐NC‐1 group, the levels of autophagy‐related proteins LC3 II/I, ATG7, and Beclin 1 were decreased, and the p62 levels was promoted in the OGD/R + sh‐METTL14 group. Following overexpression of Tp53inp2, the levels of LC3 II/I, ATG7, and Beclin 1 inreased, while the p62 level suppressed. TEM showed that the mitochondria in the OGD/R + sh‐NC‐1 group were obviously swollen, and the cristae were partially ruptured or disorganized. Interference with METTL14 reversed OGD/R‐induced changes. Overexpression of Tp53inp2 aggravated OGD/R‐induced changes (Figure 6L). In addition, we examined the effect of METTL14 inhibition and Tp53inp2 overexpression on neuronal differentiation of NSCs. Compared with the OGD/R + sh‐NC‐1 group, β‐III‐tubulin expression in the OGD/R + sh‐METTL14 group was increased. After overexpression of Tp53inp2, β‐III‐tubulin expression was decreased (Figure 6M,N). Furthermore, compared with the OGD/R + sh‐NC‐1 group, the number of dendrites, the total dendrite length, and the number of new neuronal junctions were increased in OGD/R + sh‐METTL14 group. After overexpression of Tp53inp2, the number of dendrites, the total dendrite length, and the number of new neuronal junctions were reduced (Figure 6O–Q). These results revealed that inhibition of METTL14 alleviated OGD/R‐induced mitophagy and ROS levels in NSCs by inhibiting the Tp53inp2/Ptgs2 axis.

## 4 Discussion

IS is a serious neurological disease for which treatment aims to restore blood flow as quickly as possible [25]. However, due to the limitations of the treatment window and reperfusion injury, treatment options are limited [26]. In this study, we explored a specific mechanism of Tp53inp2 in cerebral ischemia‐reperfusion injury through in vivo and in vitro experiments. We found that inhibition of METTL14 alleviated OGD/R‐induced neuronal differentiation injury in NSCs through the Tp53inp2/Ptgs2 axis. This is the first study to report on m6A‐modified Tp53inp2 in IS.

Tp53inp2 is a nuclear protein, mainly located in the nucleus, involved in cellular processes such as transcription, autophagy, and apoptosis, and regulates transcriptional activity by binding to various nuclear hormone receptors [27, 28]. When deprived of nutrients, Tp53inp2 is rapidly transferred from the nucleus to the cytoplasm and is involved in regulating macrophage autophagy [29]. After transfer from the nucleus, cytoplasmic Tp53inp2 targets the early autophagy membrane associated with LC3 and promotes autophagosome biogenesis by mediating LC3‐ATG7 interactions [30]. In addition, Tp53inp2 can promote ribosome biogenesis by promoting rRNA synthesis in the nucleolus, suggesting that TP53INP2 has a dual role in cell metabolism, both assisting anabolism in the nucleolar and stimulating catabolism outside the nucleolus [31]. Previous studies have shown that Tp53inp2 may exert beneficial effects in some disease models. For example, Sebastián et al. found that Tp53inp2 expression was downregulated in aging skeletal muscle of mice and humans. Chronic activation of autophagy by muscle‐specific overexpression of Tp53inp2 prevented sarcopenia and delayed muscle functional decline [32]. Bhattacharya et al. reported that Tp53inp2 is a direct target gene of miR‐638. Overexpression of Tp53inp2 significantly suppressed the proliferation and invasion of melanoma cells, whereas miR‐638 promoted melanoma progression by inhibiting TP53INP2, revealing a tumor‐suppressive function of Tp53inp2 as a downstream molecule of the p53 signaling pathway in melanoma [33]. Furthermore, Tp53inp2 promotes remyelination and improves cognitive function in aged mice by regulating α‐ketoglutarate production and lipid biosynthesis [34]. On the other hand, Sun et al. demonstrated in a spinal cord injury model that TP53INP2 expression was upregulated following spinal cord injury, and that knockdown of TP53INP2 inhibited both the inflammatory response and neuronal apoptosis, suggesting that TP53INP2 exacerbates injury in this context [35]. These findings indicate that TP53INP2 exhibited a significant functional duality across different disease backgrounds. Mitophagy is a mechanism for the selective elimination of mitochondria through autophagy. Mitochondria play a critical role in mediating neuronal and tissue damage following a stroke, and their functions include providing ATP to tissues, regulating oxidative metabolism during pathological process, and participating in apoptosis after stroke [36]. Once IS occurs, the damaged cells respond to this stress through mitophagy [37]. However, under the ischemic/hypoxic conditions of this study (OGD/R and MCAO models), Tp53inp2 is pathologically activated, as evidenced by the significantly upregulated expression of Tp53inp2 in injured NSCs. Knockdown of Tp53inp2 alleviated OGD/R‐induced mitophagy, excessive ROS production, and impaired neuronal differentiation. Meanwhile, transplantation of NSCs with Tp53inp2 knockdown effectively improved cognitive function recovery in MCAO model mice, further confirming that pathological activation of Tp53inp2 exacerbates ischemic injury. Therefore, we further investigate its mechanism in IS.

It has been reported that Tp53inp2 can activate autophagy during the early stage of bovine adipocyte differentiation and actively regulate adipocyte differentiation by influencing autophagy [19]. However, stringent control of mitochondrial quality and quantity through mitochondrial division and fusion, along with mitophagy, is essential for the survival and maintenance of normal neuronal function [38]. NSCs possess the capability to self‐renew and generate various neural lineage cells, including neurons and glial cells, thereby playing a critical role in treating neurological disorders [39]. The neuronal differentiation of NSCs is pivotal for brain recovery after stroke [40]. Enhancing neuronal differentiation in NSCs can significantly improve the structural and functional recovery of the brain after stroke [41]. In this study, we further found that Tp53inp2 mediates neuronal differentiation of OGD/R‐treated NSCs through the repression of mitophagy. Through further screening and validation, we selected Ptgs2 as the downstream target of the effect of Tp53inp2 in NSCs to study.

Ptgs2 is a key enzyme involved in the biosynthesis of prostaglandins. In addition, Ptgs2 is a classic pro‐inflammatory mediator in neuroinflammation and cerebral ischemia‐reperfusion injury, and its role in regulating oxidative stress and exacerbating injury has been validated by multiple studies [42, 43, 44]. Specifically, Ptgs2/PGE2 promotes the activation of PGC‐1α by activating the cAMP response element‐binding protein, thereby increasing mitochondrial biogenesis and exerting a protective effect on the heart [45]. In hepatocytes, the expression of Ptgs2 is protective against liver ischemia–reperfusion injury, reducing necrosis, lowering ROS levels, and increasing autophagy, as well as antioxidant and anti‐inflammatory responses [46]. In macrophages infected with Mycobacterium tuberculosis, the expression of Ptgs2 significantly increases, defending the body against the pathogen by modulating adaptive immunity and restoring the mitochondrial inner membrane [47]. Mitophagy, by eliminating excess and/or damaged organelles, mediates cell survival and vitality following injury/trauma and infection. Failure to clear damaged mitochondria leads to hyperactivation of inflammatory signaling pathways, resulting in the development of chronic systemic inflammation and inflammatory diseases [48]. Research has explored the induction of apoptosis in hepatocellular carcinoma by ketoconazole through the exacerbation of mitophagy, a process involving the downregulation of Ptgs2 [49]. Furthermore, inhibition of mitophagy saved proliferation stagnation by restoring Ptgs2 protein levels, canceled the elimination of mitochondria‐localized Ptgs2, and upregulated Ptgs2 mRNA levels [50]. Zhou et al. revealed that Tongqiao Huoxue decoction reduced nerve function damage after IS through the Ptgs2/NF‐κB axis [51]. These studies provide evidence of the relevance and role of Ptgs2 in mitophagy and demonstrate its potential therapeutic value in different disease models. Ptgs2 protects cells, reduces cellular damage, and enhances cellular autophagy by regulating mitochondrial function and mitochondrial biogenesis, thus playing an important role in innate immune responses. However, the mechanism of Tp53inp2 with Ptgs2 and mitophagy in IS remains unclear. Our finding that “knockdown of Tp53inp2 exerts protective effects by inhibiting Ptgs2” is highly consistent with previous reports [42, 43, 44], further supporting the rationale for this mechanism.

m6A modifications play a crucial role in the pathophysiological progression of IS [52], including oxidative stress, mitochondrial dysfunction, endothelial dysfunction, neuroinflammation, and cell death processes [53]. Tp53inp2 can mediate the autophagic degradation of ubiquitinated proteins through its ubiquitin interaction motif [54]. Huang et al. reported that Tp53inp2 is highly expressed and localized to the cytoplasm in NPM1‐mutant leukemia, and that FTO upregulates Tp53inp2 expression by reducing the m6A modification level of Tp53inp2 mRNA and enhancing its stability [12]. However, it remains unclear whether Tp53inp2 is methylated by m6A in IS. In this research, we found that METTL14 regulates the m6A modification level of Tp53inp2 mRNA, and this modification is positively correlated with its expression; through bioinformatics prediction and experimental validation, this study identified functional m6A sites on Tp53inp2 mRNA, although the downstream molecular mechanisms and site‐specific functions of this regulatory process require further investigation. Huang et al. finding is consistent with our finding that m6A modification regulates Tp53inp2 expression, thus providing literature‐based support for our mechanistic evidence. This study preliminarily demonstrated that YTHDF1 is involved in the m6A‐dependent regulation of Tp53inp2 and can bind to Tp53inp2 mRNA and affect its stability. However, the potential regulatory roles of other m6A readers (such as YTHDF2 and YTHDF3 of the YTHDF family, as well as members of the IGF2BP family) under different conditions cannot be excluded. The regulatory network of m6A modification is inherently complex; different m6A readers may participate in the regulation of Tp53inp2 expression through synergistic or compensatory mechanisms under different physiological or pathological states. The precise mode of their synergistic interaction requires further investigation. Further validation showed that inhibition of METTL14 alleviated OGD/R‐induced mitophagy and ROS levels in NSCs by inhibiting the Tp53inp2/Ptgs2 axis. Under normal physiological conditions, Tp53inp2 has no statistically significant regulatory effect on basal mitophagy, ROS levels, or differentiation capacity in NSCs. However, under ischemic stress, METTL14‐mediated m6A modification drives Tp53inp2 overexpression, which triggers pathological mitophagy and mitochondrial dysfunction, thereby impairing the differentiation capacity of NSCs. This clearly distinguishes the difference between physiological and pathological activation of the Tp53inp2/Ptgs2 axis.

In this study, the transplantation of NSCs with Tp53inp2 knockdown increased neuronal differentiation, thereby alleviating MCAO and mitigated cognitive impairments in MCAO model mice. In the in vitro cell model induced by OGD, increased mitophagy in NSCs induced increased ROS levels and a decreased in mitochondrial membrane potential, inhibiting the NSC differentiation. The development of cognitive impairment following stroke is closely related to the function of NSCs within the brain. Stroke can activate NSCs in the brain, which have the potential to repair damaged brain tissue and improve neurological function. For instance, one study pointed out that after stroke, intrinsic repair mechanisms involving damaged neurons can be observed through the stimulation of NSC activation and neuronal regeneration. Furthermore, NSCs play a significant role in neural regeneration and functional recovery after stroke [55]. Specifically, one study explored the relationship between the accelerated depletion of the hippocampal NSC pool following intracerebral hemorrhage, a type of hemorrhagic stroke, and cognitive impairment. This study revealed that after cerebral hemorrhage, iron accumulation in the hippocampus was associated with cognitive impairment; iron accumulation triggered excessive activation of NSCs, leading to the depletion of the NSC pool, reduced neurogenesis, and ultimately cognitive dysfunction [56]. These research results indicated that the development of cognitive impairment following stroke was closely related to the function of NSCs within the brain, and that maintaining the NSC pool may be of significant value in preventing cognitive dysfunction in patients with cerebral hemorrhage or other related conditions. Therefore, it can be stated that issues with NSCs within the brain may play a fundamental role in the development of cognitive impairment following stroke. The therapeutic effects of NSC transplantation in ischemic brain injury repair cannot be explained by a single mechanism; rather, they result from the synergistic action of multiple pathways and mechanisms. In addition to the neuronal differentiation mechanism that is the focus of this study, other well‐established mechanisms of NSC transplantation are likely involved in post‐ischemic brain tissue repair and functional reconstruction. These include: paracrine effects (secretion of neurotrophic factors, cytokines and other factors that improve the microenvironment of the injured area and promoting endogenous neuronal survival and repair); immunomodulation (regulation of microglial activation, alleviating cerebral inflammation, and suppressing the release of neurotoxic inflammatory factors); direct neuroprotective effects (reduction of apoptosis in damaged neurons and preserving of blood–brain barrier integrity); and promotion of the proliferation and differentiation of endogenous NSCs. These mechanisms act synergistically to collectively drive the behavioral and histological improvements observed in MCAO mice.

Although the additional experiments in this study identified the functional m6A modification sites on Tp53inp2 mRNA, validated the regulatory role of METTL14 at these sites, and established a direct link between them, certain limitations still exist. First, individual functional validation has not performed for all predicted high‐confidence m6A sites (e.g., positions 57/63, 3755, and 4194), and thus the specific contribution and weight of each site in regulating Tp53inp2 expression remain unclear. Second, the downstream molecular mechanisms through which m6A modification regulates Tp53inp2 expression have not been thoroughly investigated—for instance, which m6A reader proteins (e.g., YTH family proteins) bind to the METTL14‐mediated m6A modification and subsequently influence Tp53inp2 expression by modulating mRNA stability, translational efficiency, or other mechanisms. Third, the synergistic relationship between METTL14‐mediated m6A modification of Tp53inp2 and the previously described Tp53inp2–Ptgs2 regulatory axis has not been clarified. Therefore, the complete molecular network by which METTL14‐mediated m6A modification regulates Tp53inp2 expression requires further elucidation in future studies. The SRAMP prediction results and site‐directed mutagenesis experiments provided in this study offer clear directions for future in‐depth exploration of these scientific questions.

Similarly, although this study confirmed the specific binding of YTHDF1 to Tp53inp2 mRNA by RIP experiments and verified its regulatory effects on Tp53inp2 expression and cellular functions through knockdown experiments, certain limitations still exist. First, rescue experiments have not been performed, so it cannot be further verified whether the regulatory effect of YTHDF1 on Tp53inp2 is direct rather than indirect. Second, direct comparative experiments with other YTHDF family members (YTHDF2 and YTHDF3) and other m6A readers have not been conducted, leaving the functional differences and synergistic relationships among different readers in the m6A regulation of Tp53inp2 unclear. Third, the precise molecular mechanism by which YTHDF1 regulates the stability of Tp53inp2 mRNA after binding (e.g., whether it recruits degradation‐related complexes or translation‐regulatory proteins) has not been investigated. Therefore, the complete mechanism by which m6A readers regulate Tp53inp2 expression awaits further elucidation through rescue experiments and comparative studies of multiple readers, and the present study provides a fundamental basis for such explorations.

The core focus of this study was to investigate the mechanism by which Tp53inp2 regulates NSC differentiation and to evaluate the therapeutic efficacy of transplanting Tp53inp2‐knockdown NSCs in MCAO mice. Therefore, other potential mechanisms of NSC transplantation (such as paracrine effects and immunomodulation) were not systematically dissected or validated, and thus their contributions to in vivo functional recovery cannot be fully excluded, nor can the synergistic interactions among different mechanisms be clearly defined. Consequently, the complete molecular network underlying the therapeutic effects of transplanted NSCs in vivo awaits further systematic elucidation in future studies. Subsequent research employing multi‐omics analyses, conditional knockout approaches, and other experimental strategies should investigate the specific roles of paracrine, immunomodulatory, and other mechanisms and clarify the synergistic relationships among them, thereby providing a more comprehensive theoretical foundation for NSC transplantation as a treatment for ischemic brain injury.

Through a series of experiments, this study has preliminarily revealed the METTL14‐m6A‐Tp53inp2‐Ptgs2 regulatory axis, the regulatory role of YTHDF1, and the therapeutic effects of transplanting Tp53inp2‐knockdown NSCs in MCAO mice. However, it should be noted that most of the experimental results are primarily correlative, and direct causality has not yet been fully established through more rigorous functional validation experiments (e.g., rescue experiments and conditional knockout experiments, etc.). Therefore, all statements regarding regulatory relationships and functional associations in this manuscript are reasonable inferences based on the current experimental data, rather than absolute causal conclusions. The relevant direct causal mechanisms require further validation in future studies.

In conclusion, this study preliminarily evaluated the mechanism of m6A‐modified Tp53inp2 in IS and identified its potential mechanism related to mitophagy and ROS‐mediated neuronal differentiation of NSCs. Our results suggest a new approach to the treatment of IS. Enhanced knowledge of the intricate involvement of mitochondria in ischemia‐related neuronal death and neuroprotection could serve as a foundation for the advancement of novel therapeutic strategies for IS.

## Author Contributions

The authors confirm contribution to the paper as follows: study conception and design: Guoshuai Yang and Dan Yu; data collection: Dan Hou, Yujie Hu, Tian Yun and Guoshuai Yang; analysis and interpretation of results: Dan Yu; draft manuscript preparation: Dan Hou. All authors reviewed the results and approved the final version of the manuscript.

## Funding

This work was supported by Hainan Province Science and Technology Special Fund, ZDYF2023SHFZ105, and National Natural Science Foundation of China, 82660281.

## Ethics Statement

The animal experiments were approved by the Ethics Committee of the Central South University Xiangya School of Medicine Affiliated Haikou Hospital (No. 2023‐LL‐154).

## Consent

The authors have nothing to report.

## Conflicts of Interest

The authors declare no conflicts of interest.

## Supporting information


**Figure S1:** Isolation and identification of NSCs. (A) Optical images of Primary NSCs. (B) Primary NSCs were identified by IF. (C) Western blot detection of Tp53inp2 expression. *p < 0.05 versus sh‐NC. For the cell experiments, *n* = 3.

**Figure S2:** Altering Tp53inp2 expression has no significant effects on mitochondrial ROS, autophagy, or neuronal differentiation. (A) Western blot detection of Tp53inp2 overexpression efficiency. *p < 0.05 versus oe‐NC. (B) Flow cytometry detection of mitochondrial ROS levels. (C) Western blot detection of autophagy‐related proteins LC3 II/I, ATG7, Beclin 1, and p62. (D) IF detection of β‐III‐tubulin expression. Scale bar = 25 μm (400 ×). (E) Statistical analysis of the positive rate of β‐III‐tubulin. (F) Statistical analysis of the number of dendrites. (G) Statistical analysis of total dendrite length. (H) Statistical analysis of the number of junctions in newborn neurons. For the cell experiments, *n* = 3.

**Figure S3:** Prediction and experimental validation of m6A modification sites on Tp53inp2 mRNA. (A) SRAMP web‐based prediction of potential m6A target binding sites of Tp53inp2. Top: predicted score distribution curve of Tp53inp2 mRNA and four confidence thresholds; the four high‐confidence sites are marked. Middle: Tp53inp2 gene structure (three exons) on Chr:2 and primer positions. Bottom: sequence details of the four predicted m6A sites (GGACU or GAACU motifs). (B) RNA secondary structure prediction showing that the m6A sites (highlighted in red/yellow) are located in loop regions. Left: local structure of Site 1/2 (nt 61–71 and 55–75). Right: global and magnified structure of Site 4 (nt approximately 4194). (C) The expression of methylation‐related enzymes METTL14/16 was monitored by qRT‐PCR and Western blot. *p < 0.05 versus Control. (D and E) Transfection efficiency detection. (F and G) Tp53inp2 levels were determined via qRT‐PCR and Western blot. & *p* < 0.05 versus sh‐NC. (H) MeRIP‐qPCR detection of m6A enrichment levels in different regions of Tp53inp2 mRNA (groups: m6A‐1, covering the region of positions 57/63; m6A‐2, covering the region of position 3755; m6A‐3, covering the region of position 4194). *p < 0.05 versus IgG. (I) Schematic diagram of m6A site mutations in Tp53inp2 mRNA. (J) MeRIP‐qPCR detection of m6A enrichment in wild‐type and mutant Tp53inp2 (groups: 1. WT group (wild‐type, IgG control); 2. MUT‐1 group (Mut1 mutant, m6A antibody IP); 3. MUT‐2 group (Mut2 mutant, m6A antibody IP)). *p < 0.05 versus IgG. (K and L) Transfection efficiency detection. & *p* < 0.05 versus sh‐NC. For the cell experiments, *n* = 3.

**Table S1:** The primer used in this study.

**Data S1:** Supporting Information.

## Data Availability

The data that support the findings of this study are available from the corresponding author upon reasonable request.
