# Supplementary material for: Inhibition of METTL14 Alleviates OGD/R‐Induced Neuronal Differentiation Injury in Neural Stem Cells Through Suppressing the Tp53inp2/Ptgs2 Axis: Potential Benefits for the Mouse MCAO Model
Source: CNS Neurosci Ther. 2026 Sep 1;32(9):e71099. doi: 10.1002/cns.71099 (PMC13535141; doi:10.1002/cns.71099)
Supplement: Supplementary file 1 [file CNS-32-e71099-s002.docx]

**Supplementary Table 1. The primer used in this study.**

| **Name** | **Sequences** |
| --- | --- |
| M-Tp53inp2-F | TTTCATTCCTGGCAATTCCCT |
| M-Tp53inp2-R | TCAAAACCCATGCCTGATCCC |
| M-YTHDF1-F | TCTGCCACTGCATGAGGTTT |
| M-YTHDF1-R | GTCGCAAAGCTGAACCAGAC |
| M-METTL14-F | GACTGGCATCACTGCGAATG |
| M-METTL14-R | AGGTCCAATCCTTCCCCAGA |
| M-METTL16-F | GAGCGTAGCATCTGCGTTTC |
| M-METTL16-R | CTTAGAGCCATCTCGCAGGC |
| M-β-actin-F | ACATCCGTAAAGACCTCTATGCC |
| M-β-actin-R | TACTCCTGCTTGCTGATCCAC |
