# Supplementary material for: Inhibition of METTL14 Alleviates OGD/R‐Induced Neuronal Differentiation Injury in Neural Stem Cells Through Suppressing the Tp53inp2/Ptgs2 Axis: Potential Benefits for the Mouse MCAO Model
Source: CNS Neurosci Ther. 2026 Sep 1;32(9):e71099. doi: 10.1002/cns.71099 (PMC13535141; doi:10.1002/cns.71099)
Supplement: Supplementary file 2 [file CNS-32-e71099-s005.docx]

**Supplementary Materials and Methods**

**Construction of middle cerebral artery occlusion (MCAO) model**

C57BL/6 mice (20-25 g) were procured from Hunan SJA Laboratory Animal Co., LTD. The mice were given one week to acclimate in a temperature-controlled animal facility with a 12 h light/dark cycle and were given standard rodent food during this period. Mice were randomly divided into the following groups: Sham, MCAO, MCAO + NSCs, MCAO + NSCs^sh-NC-1^, and MCAO+NSCs^sh-Tp53inp2^, with 12 mice in each group. The MCAO model was induced following established procedures as described in previous studies [1, 2]. After anesthesia, mice were supine fixed. The neck hair of mice was sterilised. A longitudinal incision of 1.5 cm was performed at the center of the neck to expose the left common carotid, internal carotid, and external carotid arteries. The thrombus was carefully advanced into neck through external carotid artery to a distance of approximately 8 mm, occluding the blood vessel for 60 minutes. Subsequently, the thrombus was withdrawn to allow for blood reperfusion. In Sham group, only internal and external carotid arteries were separated. Referring to previous studies [3-5], mice were anesthetized and placed in a mouse stereotactic instrument (RWD, China). A burr hole was drilled in the skull, and a stab incision was made above the lateral ventricle (AP+0, ML-1, DV-2.25 mm) for the injection of NSCs. Twenty-four hours after reperfusion in MCAO mice, 5 μL of 5 × 10^5^ NSCs, either untreated or transfected with sh-NC-1 or sh-Tp53inp2, were injected in PBS solution. The modified neurological severity score (mNSS) was performed at 0, 1, 3, 7, and 14 days after treatment. Based on previous studies reported protocols, behavioral tests (the balance beam, rotarod tests, and ladder lung) [3] were performed at 0, 1, 3, 7, and 14 days after treatment. Tissue collection was performed 14 days after ischemia-reperfusion, with one whole brain from each group being used for 2,3,5-Triphenyltetrazolium Chloride (TTC) staining, and the remaining mice were subjected to whole brain fixation.

**Assessment of mNSS**

Neurological deficits in mice were evaluated using mNSS, which encompasses assessments of motor function, sensory function, beam balance, and reflex abnormalities. Total score ranges from 0 to 18, with higher scores correlating with more severe damage. The mNSS scores for each group of mice were recorded 24 h after the ischemic procedure [1].

**TTC staining**

TTC was utilized to measure infarction size. Mouse brain tissue was taken and sectioned (5 slices) at 2 mm intervals. Brain slices were immersed in TTC solution, covered with aluminum foil, and then incubated for 15-30 min at 37°C. The slices were periodically inverted to ensure uniform contact with the staining solution.

**Primary cell extraction and model construction**

Primary NSCs were cultured as described in previous studies [6]. Primary NSCs were extracted from the fetal mice cortex on day 16 of an embryo. The cortical tissue was mechanically fragmented into small pieces until a cloudy suspension was achieved. Subsequently, suspension was transferred to a new tube and centrifuged at 600 g for 2 min. Following centrifugation, particles were re-suspended in a culture flask at a concentration of 1×10^6^ cells/mL. NSCs were cultured in Dulbecco’s Modified Eagle’s Medium (DMEM)/F12 medium supplemented with 20 ng/mL of basic fibroblast growth factor, 20 ng/mL of epidermal growth factor, 2% B27 supplement, and 1% penicillin/streptomycin. Once a nerve ball with a diameter of 100 μm was formed, NSCs were passed through enzyme separation every 4-6 days. In this study, NSCs from the 2nd to 5th generation were utilized. With the exception of the oxygen-glucose deprivation (OGD) experiment, all cultures were maintained in cell incubators at 37°C, 5% CO_2,_ and 95% air. As previously mentioned, primary NSCs were first transfected with sh-NC, oe-NC, sh-NC-1, oe-NC-1, sh-Tp53inp2, oe-Prostaglandin Endoperoxide Synthase 2/Cyclooxygenase 2 (Ptgs2/COX2), sh-METTL14 or oe-Tp53inp2 [7], then OGD/reperfusion (OGD/R) model was constructed. OGD/R model was established following protocols from previous literature [2]. Briefly, NSCs were cultured in a glucose-free medium and exposed to a gas mixture (1% O_2_, 5% CO_2_, and 94% N_2_) at 37°C. After 3 h of OGD, NSCs were subsequently placed in a normal medium and maintained in a standard-oxygen incubator, and cultured in a differentiation medium for 5 days to promote cell differentiation [6]. On the last day of differentiation culture, 300 nM rapamycin was treated for 24 h [8].

**Morphological analysis of newborn neurons**

For morphological assessment, neurons were reconstructed using Imaris 7.3.0 (Bitplane Scientific Software). The Image J Sholl Analysis Plugin (NIH, Bethesda) was employed for analysis. The nucleus of the cell body served as the center of all concentric circles. Starting and ending radii were set at 10 mm and 100 mm from the center, respectively, with a 10 mm interval between successive radii. Each neuron’s complexity was evaluated by counting the number of intersection points between concentric circles and dendrites, as well as by summing the lengths of all dendrites to determine the total dendrite length. Higher intersection counts or total dendrite lengths indicated more intricate dendritic branching in the neurons [9].

**Immunofluorescence (IF)**

Nestin and β-III-tubulin expressions in primary NSCs was assessed through IF. The crawling tablets were removed. They were then fixed with 4% paraformaldehyde for 30 min, and permeabilized with 0.3% Triton X-100 at 37°C for 30 min. 5% BSA was used to close at 37℃ for 60 min. Droplets of mono-antibody β-III-tubulin (ab78078, 1:100, Abcam) or Nestin (19483-1-AP, 1:50, Proteintech) were added at 4℃ overnight. 50-100 μL Goat Anti-Mouse IgG(H+L) Secondary Antibody (AWS0004c, 1:200, Abiowell) or Goat Anti-Rabbit IgG(H+L) Secondary Antibody (AWS0005c, 1:200, Abiowell) was incubated. DAPI working solution was applied for nuclear staining at 37°C for 10 min. The buffer glycerine was sealed and observed under a fluorescence microscope (BA410T, Motic).

Furthermore, the expression of NeuN in mouse brain tissue was examined by IF. Tissue slices were baked at 60°C for 12 h, and dewaxed to water. Tissue slices were immersed in 0.01 M citrate buffer (pH=6.0) and heated in an electric or microwave oven until boiling. After cooling, slices were washed with 0.01 M PBS (pH=7.2-7.6). Subsequently, slices were placed in a sodium borohydride solution for 30 min. The sections were placed in a 75% ethanol solution at room temperature for 5 min, then placed in Sudan black staining solution at room temperature for 5 min, followed by rinsing with water for 3 min. 10% normal serum or 5% BSA was closed for 1 h. Appropriately NeuN (AWA10318, 1:400, Abiowell) antibody was added overnight at 4℃. Secondary antibody was incubated at 37℃ for 90 min. The nuclei were stained with DAPI working solution at 37°C for 10 min, followed by rinsing with PBS for 5 min, repeated three times. The sections were then mounted with buffer glycerin, stored in the dark, or examined under a fluorescence microscope.

Additionally, the expression of DCX and BrdU in mouse brain tissue was examined by IF. Tissue slices were baked at 60°C for 12 h, and dewaxed to water. Tissue slices were immersed in 0.01 M citrate buffer (pH=6.0) and heated in an electric or microwave oven until boiling. After cooling, slices were washed with 0.01 M PBS (pH=7.2-7.6). 0.3% hydrogen peroxide was closed for 15 min. 10% normal serum or 5% BSA was closed for 1 h. Appropriately DCX (ab207175, 1:200, Abcam) antibody was added overnight at 4℃. HRP secondary antibody was incubated at 37℃ for 31 min. TYP-520 fluorescent dye was incubated at 37℃ for 5-10 min, protected from light, and washed with PBS. Then, tissue slices were immersed in 0.01 M citrate buffer (pH=6.0) and heated in an electric or microwave oven until boiling. After cooling, slices were washed with 0.01 M PBS (pH=7.2-7.6). 0.3% hydrogen peroxide was closed for 15 min. 10% normal serum or 5% BSA was closed for 1 h. Appropriately BrdU (66241-1-Ig, 1:200, Proteintech) antibody was added overnight at 4℃. HRP secondary antibody was incubated at 37℃ for 31 min. TYP-570 fluorescent dye was incubated at 37℃ for 5-10 min, protected from light, and washed with PBS. The nuclei were stained with DAPI working solution at 37°C for 10 min, followed by rinsing with PBS for 5 min, repeated three times. The sections were then mounted with buffer glycerin, stored in the dark, or examined under a fluorescence microscope.

**Quantitative real-time PCR (qRT-PCR)**

The expression of Tp53inp2, METTL14, METTL16, and YTHDF1 genes was detected by qRT-PCR. First, Trizol total RNA extraction kit (15596026, Thermo) was utilized to extract total RNA, followed by determining the concentration and purity of extracted RNA samples. Then, mRNA reverse transcription kit (CW2569, CWBIO) was applied to reverse transcription mRNA into cDNA. Ultra SYBR Mixture (CW2601, CWBIO) was employed to examine genes expression on ABI 7900 system. Primers are shown in supplementary Table 1. mRNA expression level was calculated by the 2^-∆∆Ct^ method with β-actin as reference gene.

**Western blot**

The expression of TP53inp2, LC3, ATG7, Beclin1, p62, Ptgs2, Hmga1, and methylation-related enzymes 14/16 in cells was analyzed using Western blot. Initially, samples from different groups were treated with RIPA (AWB0136, Abiowell) to extract total proteins. These proteins were then separated by SDS-PAGE and transferred to NC membrane. Subsequently, membrane was blocked with 5% skim milk and then incubated with primary antibodies overnight at 4°C. Primary antibodies include: TP53inp2 (AWA51376, 1:1000, Abiowell), LC3 (AWA55385, 1:1000, Abiowell), ATG7 (AWA41926, 1:1000, Abiowell), Beclin1 (AWA50785, 1:1000, Abiowell), p62 (18420-1-AP, 1:4000, Proteintech), Ptgs2 (ab179800, 1:3000, Abcam), Hmga1 (AWA43961, 1:1000, Abiowell), METTL 14 (ab300104, 1:1000, Abcam), METTL 16 (19924-1-AP, 1:600, Proteintech), and β-actin (AWA80001, 1:5000, Abiowell). Goat anti-mouse IgG (H+L) Secondary Antibody (AWS0001, 1:5000, Abiowell) or Goat anti-rabbit IgG (H+L) Secondary Antibody (AWS0002, 1:5000, Abiowell) was incubated. Membrane was incubated with ECL reagent (AWB0005, Abiowell) for 1 min and subjected to imaging system analysis. β-actin was utilized as reference protein, and each protein levels were analyzed with Quantity One 4.6.6.

**Measurement of lactate dehydrogenase (LDH) levels**

LDH levels were determined with LDH kit (A020-2, Nanjing Jiancheng Bioengineering Institute) as per the provided instructions.

**Detection of mitochondrial membrane potential**

Cells were centrifuged at 1000 rpm for 5 min. Preparation of JC-1 staining working solution (C2006, Beyotime): The required volume of JC-1 staining working solution for each well of a six-well plate was 1 mL, and a similar amount was used for other culture vessels. For cell suspension, 0.5 mL of JC-1 staining working solution was needed for every 500,000-1,000,000 cells. An appropriate amount of JC-1 (200×) was taken and JC-1 was diluted by adding 8 mL ultra-pure water per 50 µL JC-1 (200×). Then 2 mL of JC-1 dyeing buffer (5×) was added and mixed to create JC-1 dyeing working solution. Subsequently, 1 mL of JC-1 dyeing solution was added and thoroughly mixed. Cells were incubated at 37°C for 20 min. Meanwhile, an appropriate volume of JC-1 dye buffer (1×) was prepared via diluting 4 mL of distilled water with each 1 mL of JC-1 dye buffer (5×) and placed in an ice bath. After the 37°C incubation, supernatant was removed, and cells were washed twice with JC-1 dyeing buffer (1×). Finally, an adequate amount of cell culture medium was added for flow cytometry (A00-1-1102, Beckman).

**Detection of ROS levels**

50 μg MitoTracker Green FM powder was fully dissolved with 13 μL DMSO to form a 5 mM storage solution, and diluted 1000 times to form a 5 μM working solution. The cells were detached and harvested using pancreatic enzymes. Subsequently, cells were pelleted once, and centrifuged at 1500 rpm for 5 min. Cells were gently re-suspended with MitoTracker Green FM solution preheated at 37℃ and incubated at 37℃ for 15-30 min. Cells were pelleted, and centrifuged at 1500 rpm for 5 min. Subsequently, ROS levels were evaluated using flow cytometry.

**Transmission electron microscopy (TEM)**

The changes in mitochondria and autophagosomes were evaluated using TEM. Cells were fixed with 2.5% glutaraldehyde (DF0156, Leagene) and osmium tetroxide (18456, TED PELLA INC) for 2 h. Following salt reduction treatment, the cells were dehydrated with ethanol and acetone. Subsequently, cells were incubated with propylene oxide: epoxy resin (1:1 ratio) for 1-2 h, followed by immersion in pure epoxy resin for an additional 2-3 h. Samples were then embedded in pure epoxy resin and polymerized in an oven at 40°C for 12 h. The resin blocks were sectioned and further baked at 60°C for 48 h. Embedding block was taken and cut into ultra-thin sections. A copper net was used to retrieve the slices. The slices were electronically stained (lead and uranium staining) and photographed with a JEM-1100 TEM (JEOL, Japan).

**Hematoxylin-eosin (HE) staining**

HE staining was utilized to evaluate brain morphological lesions. Brain tissue was taken from mice and subsequently underwent embedding, dewaxing, and sectioning. The tissue was stained with hematoxylin (AWI0009, Abiowell) and eosin (AWI0020, Abiowell). Following staining, the sections were immersed in xylene solution and mounted with neutral gum. The brain morphology was observed by microscope.

**Proteomic analysis**

Tp53inp2 was overexpressed and divided into oe-NC and oe-Tp53inp2 groups for proteomic analysis. Based on Raw files assessed by mass spectrometry, a sample-specific protein database was constructed according to sample source. Subsequently, analysis software was employed to search constructed database. Quality control analysis of peptide and protein levels was conducted based on database search results. Principal component analysis (PCA) was performed using relative quantitative values of all samples, and a visual PCA map was generated. Clustering was carried out according to the similarity degree of protein expression trend. In addition, the common functions of the identified proteins were annotated. In DAVID version 6.8 (http://david.abcc.ncifcrf.gov), differentially expressed proteins were analyzed in terms of Kyoto Encyclopedia of Genes and Genomes (KEGG) pathways.

**Co-immunoprecipitation (Co-IP)**

To investigate the interaction between Tp53inp2 and Ptgs2, we conducted a Co-IP assay with Ptgs2 antibody as the bait protein. First, cells are collected for protein extraction. Subsequently, the extract containing the same amount of protein was immunoprecipitated in a lysate buffer containing Ptgs2 antibodies overnight at 4°C. Protein A/G agarose beads were added to the immunoprecipitation mixture and allowed to incubate for 2 h. The immunoprecipitated complex was then eluted in an SDS-PAGE sample buffer, boiled, separated by SDS-PAGE, and transferred to a NC membrane. Protein was first incubated with Tp53inp2 and Ptgs2, and then with the corresponding secondary antibody. The membrane was incubated with ECL reagent and subjected to analysis using an imaging system.

**RNA immunoprecipitation (RIP) assay**

YTHDF1 (17479-1-AP, Proteintech), YTHDF2 (24744-1-AP, Proteintech), and YTHDF3 (ab220161, Abcam) proteins were tested for binding to trp53inp2 with EZMagna RIP kit (Millipore, USA). In short, cells were lysed at 4°C for 30 min with RIP lysis buffer and subsequently incubated with RIP buffer containing magnetic beads conjugated to antibodies against Ago2 (CST, USA) or anti-rabbit IgG (negative control, CST, USA). The retrieved RNA was subjected to qRT-PCR analysis, with total RNA serving as input controls.

**m6A-RIP**

Cellular RIP was performed to quantitatively measure the m6A modification levels of METTL14 and Tp53inp2 using the antibody (68055-1-Ig, Proteintech). Cells were collected and lysed using a lysis buffer containing protease and phosphatase inhibitors to ensure the integrity of RNA-binding proteins. The cell lysates were incubated overnight at 4°C with an antibody specifically recognizing m6A modification to allow the antibody to bind to RNA-binding proteins containing m6A modification. The immunocomplexes were then incubated with protein A/G beads for 2 at 4°C to capture the RNA-binding proteins bound by the antibody. The beads were washed multiple times with a wash buffer containing RNA stabilizers to remove unbound proteins and non-specific binders. The beads were then incubated with an RNA release buffer at a high temperature to release the RNA co-precipitated with the RNA-binding proteins. RNA was extracted from the supernatant using an RNA purification kit. qPCR reactions were performed using specific primers for METTL14 and Tp53inp2. The primer sequences are as follows: M-Trp53inp2-F: TTTCATTCCTGGCAATTCCCT, M-Trp53inp2-R: TCAAAACCCATGCCTGATCCC; M-METTL14-F: GACTGGCATCACTGCGAATG, M-METTL14-R: AGGTCCAATCCTTCCCCAGA.

**Prediction of m6A modification sites on Tp53inp2 mRNA**

The potential m6A modification sites in Tp53inp2 mRNA were predicted using the sequence‑based RNA adenosie methylation site predictor (SRAMP) online tool (http://www.cuilab.cn/sramp). The analysis was performed using the random forest model with the default settings.

**MeRIP‑qPCR validation of predicted m6A sites**

To validate the predicted m6A sites, MeRIP‑qPCR was performed. Total RNA extracted from cells were fragmented to 100–200 nt. A portion was saved as input control, and the remainder was incubated with anti‑m6A antibody or control rabbit IgG. RNA‑antibody complexes were captured using protein A/G magnetic beads, and bound RNA was eluted. Enrichment of Tp53inp2 mRNA fragments was detected by qPCR using SYBR Green. Primers were designed to cover three regions containing the four high‑confidence m6A sites predicted by SRAMP: m6A‑1 (positions 57/63): forward 5’-CAGACTGGCCTTTAACTC-3’, reverse 5’-TATGACAGGGTGCTTTGG-3’; m6A‑2 (position 3755): forward 5’-TATGTGCCTTTCACCTTG-3’, reverse 5’-CTCTAACCTCCTCCTGTC-3’; m6A‑3 (position 4194): forward 5’-TGCAACAAGGACCCTACCA-3’, reverse 5’-ACCCAACAGAGATGCACTCAA-3’. The experimental groups were m6A‑1, m6A‑2, and m6A‑3. Enrichment fold was calculated as the ratio of m6A antibody‑pulled RNA to IgG control‑pulled RNA, normalized to input.

**Site‑directed mutagenesis of putative m6A sites**

To further clarify the regulatory role of specific m6A loci in Tp53inp2 mRNA methylation, we conducted site-directed mutagenesis targeting core m6A modification sites. The mutagenesis strategy was as follows: Mut1 introduced an A‑to‑G substitution at the region spanning positions 4194, and Mut2 introduced an A‑to‑T substitution at another high‑confidence site.

**MeRIP‑qPCR validation of mutant m6A sites**

To validate the functional contribution of specific m6A sites, wild‑type (WT) and mutant sequences corresponding to the predicted m6A region of Tp53inp2 were synthesized and inserted into a plasmid backbone. The inserted fragment (approximately 200 bp) contained the predicted m6A site (positions 57/63) with the following core motifs: WT (5’-GGACT-3’), MUT1 (5’-GGGCT-3’; A‑to‑G substitution), and MUT2 (5’-GGTCT-3’; A‑to‑T substitution). Cells were transfected with the WT, MUT1, or MUT2 construct. MeRIP‑qPCR was performed as described above using an anti‑m6A antibody or control IgG. Enrichment of the targeted region was detected by qPCR using the following specific primers (M‑Trp53inp2): Forward: 5’-TGCAACAAGGACCCTACCA-3’, Reverse: 5’-ACCCAACAGAGATGCACTCAA-3’. The following experimental groups were included: WT group (cells transfected with wild‑type construct, IgG control), MUT‑1 group (cells transfected with Mut1 mutant, m6A antibody IP), and MUT‑2 group (cells transfected with Mut2 mutant, m6A antibody IP). The enrichment of m6A in the targeted regions was compared between WT and mutant groups.

**Tp53inp2 mRNA stability detection**

Tp53inp2 mRNA stability was detected after actinomycin D treatment. The method for extracting total RNA utilized in this study for PCR examination followed a previously established protocol. Ultimately, the mRNA expression levels at the designated time points were determined and standardized relative to β-actin [10].

**References**

1.	Cai, Y., et al., *FUNDC1-dependent mitophagy induced by tPA protects neurons against cerebral ischemia-reperfusion injury.* Redox Biol, 2021. **38**: p. 101792.

2.	Wang, M., et al., *Homocysteine enhances neural stem cell autophagy in in vivo and in vitro model of ischemic stroke.* Cell Death Dis, 2019. **10**(8): p. 561.

3.	Zhang, R., et al., *NSC-derived exosomes enhance therapeutic effects of NSC transplantation on cerebral ischemia in mice.* Elife, 2023. **12**.

4.	Zhu, J.D., et al., *Effects of Noggin-Transfected Neural Stem Cells on Neural Functional Recovery and Underlying Mechanism in Rats with Cerebral Ischemia Reperfusion Injury.* J Stroke Cerebrovasc Dis, 2017. **26**(7): p. 1547-1559.

5.	Tang, Y., et al., *Neural stem cell protects aged rat brain from ischemia-reperfusion injury through neurogenesis and angiogenesis.* J Cereb Blood Flow Metab, 2014. **34**(7): p. 1138-47.

6.	Zhang, F., et al., *Phosphofructokinase-1 Inhibition Promotes Neuronal Differentiation of Neural Stem Cells and Functional Recovery After Stroke.* Neuroscience, 2021. **459**: p. 27-38.

7.	Dong, S., J. Li, and X. Zhang, *Tumor protein p53-induced nuclear protein 2 modulates osteogenic differentiation of human adipose derived stem/stromal cells by activating Wnt/β-catenin signaling.* Am J Transl Res, 2020. **12**(10): p. 6853-6867.

8.	Maity, J., et al., *KLF2 regulates dental pulp-derived stem cell differentiation through the induction of mitophagy and altering mitochondrial metabolism.* Redox Biol, 2020. **36**: p. 101622.

9.	Zhang, W., et al., *TP53INP2 Promotes Bovine Adipocytes Differentiation Through Autophagy Activation.* Animals (Basel), 2019. **9**(12).

10.	Wang, X., et al., *RBM15 facilitates laryngeal squamous cell carcinoma progression by regulating TMBIM6 stability through IGF2BP3 dependent.* J Exp Clin Cancer Res, 2021. **40**(1): p. 80.
